# The Effect of Prophylactic Anticoagulation with Heparin on the Brain Cells of Sprague-Dawley Rats in a Cardiopulmonary-Cerebral Resuscitation Model

**DOI:** 10.1155/2020/8430746

**Published:** 2020-09-20

**Authors:** Wenxun Liu, Yun Wang, Xiaohong Zhou, Kerong Hai, Danting Jia, Qingshan Ye

**Affiliations:** ^1^Ningxia Medical University, Yinchuan, China; ^2^Department of Anesthesiology, People's Hospital of Ningxia Hui Autonomous Region, Yinchuan, China

## Abstract

After a cardiac arrest (CA) of 5 to 10 min, a marked activation of blood coagulation occurs and microthrombi are found in the cerebral vessels. These microcirculatory disturbances directly affect the outcome on cardiopulmonary resuscitation (CPR). The purpose of this study was to investigate the effects and potential mechanisms of prophylactic anticoagulation on rat brain cells after cerebral CPR. After setting up an asphyxial CA model, we monitored the basic parameters such as the vitals and survival rate of the rats and assessed the respective neurological deficit (ND) and histological damage (HD) scores of their brain tissues. We, furthermore, investigated the influence of heparin on the expressions of TNF-*α*, IL-1*β*, CD40, NF-*κ*B, and HIF-1*α* after asphyxial CA. The results showed that anticoagulation with heparin could obviously improve the outcome and prognosis of brain ischemia, including improvement of neurological function recovery and prevention of morphological and immunohistochemical injury on the brain, while significantly increasing the success rate of CPR. Treatment with heparin significantly inhibited the upregulation of CD40, NF-*κ*B, and HIF-1*α* induced by asphyxial CA. Thrombolysis treatment may improve the outcome and prognosis of CPR, and future clinical studies need to evaluate the efficacy of early heparin therapy after CA.

## 1. Introduction

Cardiac arrest (CA) induces whole-body ischemia, which causes damage to multiple organs, including the brain, heart, kidney, and liver [1]. How to reduce damages to these organs in CA is critical to developing better resuscitation strategies to improve patient survival rates, which has not improved over the last few decades [2]. Reducing brain injury is a key issue following cardiopulmonary resuscitation (CPR). After CPR, ischemia-reperfusion injury can cause a series of changes, such as tissue and vascular endothelial cell damage, activation of the coagulation system, and decreased tissue plasminogen activator. Brain damage after CA is typically the result of ischemic or hypoxic injury in vulnerable areas of the brain such as the hippocampus, cortex, and thalamus and triggers a series of pathophysiological processes following CA/CPR. The necrosis and apoptosis of numerous nerve cells lead to a variety of neuronal dysfunctions, including anterograde amnesia, learning difficulties, emotional and social behavioral changes, depression, potentially coma, persistent vegetative state, and death [3]. Therefore, brain damage has been recognized as a major sign of post-CA syndrome, which usually increases mortality in addition to the primary diseases. Effective attenuation of brain injury is one of the key aims of cardiopulmonary-cerebral resuscitation (CPCR).

Adverse events during CA and CPR are characterized by excessive coagulation, inadequate endogenous anticoagulation, and fibrinolysis, as well as an inflammatory syndrome that closely resembles the immunological profile observed in sepsis patients [4]. Nevertheless, anticoagulation or thrombolysis was contraindicated during CPR for many years due to the fear of severe bleeding complications. Case reports using thrombolysis showed significantly improved survival for patients after thrombolysis during CPR. Other trials showed that bleeding complications do not occur more frequently after thrombolysis during CPR. Experimental investigations demonstrated that thrombolysis during CPR improves cerebral microcirculation [5, 6].

CD40 is a type I phosphoprotein with a molecular weight of 50 kD, belonging to the tumor necrosis factor (TNF) receptor superfamily. CD40 is mainly expressed at the surface of B Cells, dendritic cells, and monocytes and is also an important signal recognition receptor for activation and maturation of T and B cells [7, 8]. The dysfunction of the CD40/CD40L system has been reported to participate in the development and progression of atherosclerosis, sepsis, and other inflammatory diseases [8–11]. In the brain, CD40 is mainly expressed in the microglia, neurons, and neuron-like cells [12]. When CD40 binds to CD40L, it may activate a variety of signaling pathways including NF-*κ*B, leading to changes in the expression and function of many procoagulant and proinflammatory genes. So, the costimulatory CD40/CD40L receptor/ligand dyad serves as a well-known link of thrombogenesis and inflammation. Hypercoagulability and inflammation may lead to the production of various inflammatory cytokines and neuronal toxins, further aggravating the inflammatory response in brain tissue after CA. Therefore, in this study, we have conducted experiments on rats to investigate the effect of prophylactic anticoagulation on the brain cells after CPCR and to explore whether it may play a protective role by regulating the level of CD40. Our main goal is to improve post-CA brain injury management.

## 2. Materials and Methods

### 2.1. Subjects

A total of 70 healthy male Sprague Dawley (SD) rats, aged 8-9 months and weighing 350–450 g, were well fed according to laboratory experiment requirements one week before the experiment, with free access to water in the cages, whereby the temperature was maintained between 15–25 degrees at a humidity rate of 45–55%.

All animal procedures were approved and conducted in accordance with the Animal Ethics Committee of NingXia Medical School and the National Animals Scientific Procedure Regulations. Animals were provided by the Laboratory Animal Center of NingXia Medical Center.

### 2.2. Grouping

The SD rats were randomly divided into 4 groups: the normal control group (*n* = 10 rats without anesthesia and operation), the sham group (*n* = 10 rats with anesthesia only), the nonanticoagulation group (*n* = 25 rats with anesthesia and CA/CPCR), and the anticoagulation group (*n* = 25 rats with anesthesia and operation with prophylactic heparinization before CA) [13].

During the course of the experiment, there were a total of 9 subjects excluded. There were three cases of liver injury complicated with massive hemorrhage, one case of lung injury, three cases of vascular injury with blood loss of more than 1.5 ml, and 2 cases of inadequate ventilation and insufficient oxygen supply. Eventually, there were 10 subjects in the control and sham groups and 25 subjects in the nonanticoagulation and heparin groups. There was no significant difference in the weights among the 4 groups before the start of the experiment.

There was no difference in the weights before and after the experiment in the subjects of the sham group (Table 1). However, asphyxial CA caused a significant decrease in weights in nonanticoagulation and heparin groups after the experiment. But, there was no significant difference between the two groups (Table 1). There were also no significant differences in the baseline MABP, HR, RT, and P_E_tCO_2_ between nonanticoagulation and heparin groups (Table 2).

### 2.3. Asphyxial CA Model

In order to setup the respiration-CA model, the subjects were stabilized for 5–8 min, and then, the baseline MABP, HR, TR, PEtCO2, and HR were recorded before another intravenous dose of 1 mg/Kg vecuronuim and after 1 min, mechanical ventilation was stopped, the subject was extubated, and the tracheal catheter was clipped to induce asphyxia. 2–4 min after asphyxia, there would be a brief episode of elevated blood pressure followed by a drop in pressure to null pressure, MABP ≤10 mmHgl. At the same time, there would have been an episode of progressive tachycardia, which would eventually slow down until CA. The electrocardiogram would show static ECG, ventricular fibrillation, and electrical mechanical dissociation. The apical region heart beats would disappear with cyanosis of the lips and skin mucosa followed by a gradual drop in the rectal temperature.

### 2.4. CPR

8 min after asphyxia, the clipped trachea catheter is opened, and then, 4-5 times of fast ventilation of pure oxygen at a tidal volume of 8–10 ml was performed and, then, maintained at a tidal volume of 1 ml at a mechanical ventilation rate of 60–75 times/min. 0.01 g/kg adrenaline followed by 1 mmol/L.kg NaHCO_3_ were administered intravenously. At the same time, CPR was performed with cardiac compression with one horizontal finger at the level of the xiphoid process and two fingers on the lateral sides of the sternum at a frequency of 200 beats per minute until independent arterial blood pressure >60 mmHg and restoration of spontaneous circulation (ROSC). The standards of resuscitation: ECG showed normal QRS waves, palpation of obvious heart throb, cyanosed skin and lips turn rosy, and a mean arterial pressure greater than 60 mmhg. Therefore, a controlled asphyxia of 8 min can definitely cause a pulseless period of 4-5 min (CA). After ROSC, high-frequency ventilation of 60–80 times/min is maintained for 10 min, and then, the ventilation frequency is adjusted to maintain a PEtCO2 of 30–35 mmHg.

### 2.5. Postresuscitation Management

Mechanical ventilation is maintained for 60 min after ROSC, and there is no need to use muscle relaxants after spontaneous respiration returns. There is no requirement to use vasosuppressors when MABP <60 mmHg, and slow intravenous administration of isotonic saline at 2-3 ml/kg would maintain normal MABP. 60 min after stopping the mechanical ventilation, the trocars are removed and the blood vessels are clamped and the skin is sutured. At this time, 75–80% oxygen is supplied using the “T”-shape tube. When the breathing rate is greater than 60 beats/min and less than 120 beats/min, the pharyngeal and corneal reflexes would recover, and there would not be any bradycardia (HR < 300 beats/min). After breathing atmospheric air without abnormal findings for 5 min, extubation is performed.

After extubation, the rats are kept in the prone position into a box with 50% oxygen for 30 min and, then, returned to the dark and quiet cages with air to breathe and 12-hour day and night alternation. During 60–120 min after ROSC, an isotonic saline solution is administered hypodermically at a dose of 20 ml/kg to prevent dehydration, and this procedure is repeated once a day, until the rats can feed on water.

In case of any of the following events, the subject would be excluded from the study: bleeding of more than 1.5 ml due to the operation; injury other than the surgery (respiratory tract, lung, and liver damage); and inadequate ventilation or limited oxygen supply. If the overall CPR time exceeds more than 2 min, resuscitation is considered to be failed. The subjects surviving for less than 72 h after CPR have to be excluded from the neurological deficit (ND) and histopathological damage (HD) score assessment since there would be no substantial pathological damage and behavioral problems. The ND score of the 4 groups before and 72 h after the experiment was assessed according to the rats asphyxia model ND scoring criteria by Laurence Katz et al. After assessing the 72 h ND score, the 4 groups of rats were anesthetized by an intraperitoneal injection of sodium 45 mg/Kg followed by intubation and mechanical ventilation. A left sternal thoracotomy was performed and using a 20 D trocar, and 5 ml of 10% paraformaldehyde + 1 ml of 25% glutaraldehyde + 25 ml of 0.2 M phosphate buffer + 19 ml of double distilled water were infused in the aorta, allowing for 50 ml of cerebral perfusion. Then, the brain tissues were harvested and placed in 3% paraformaldehyde (PH 7.4, 4 degrees Celcius) for 24 hours and, then, embedded in paraffin. According to the anatomical Atlas of the rat brain by Paxinos [14], the coronal presentation was prepared using 6 *μ*m paraffin sections across the 19, 29, 36, 42, and 63 regions, and then, Nissl's staining was performed. The extent of histopathological damage of the hippocampus (CA1-3) was, then, observed under microscopy. Eventually, using the Laurence Katz HD scoring system and standards, the histological damage in the hippocampus, cortex, thalamus, shell caudate nucleus, and cerebellum was assessed and scored. After cerebral perfusion, two rats were taken from each group, and the hippocampus (CA1-3) was retrieved from the same area and prepared into 1 mm^3^ electron microscopy slides.

### 2.6. Light Microscope Sample Preparation

The brain tissue was fixed in 10% neutral formalin (24 h). After fixation, the brain tissue was passed through an ethanol gradient for dehydration and, then, soaked in xylene and embedded in paraffin followed by slicing. Then, the sample was dewaxed in xylene and rehydrated through decreasing concentrations of ethanol and stained with HE.

After rehydration with ethanol, the samples were sectioned, and then, immunohistochemical staining was performed and the slice was mounted. The rate of immunohistochemical expression of TNF-*α*, IL-1 in the neurons of the hippocampus (CA1-3) was statistically analyzed to assess the extent of brain histological damage (the TNF-*α*, IL-1*β* reagents were provided by the Wuhan Ph.D. Biological Engineering Co., Ltd.).

### 2.7. Sample Preparation for Electron Microscopy

1 mm^3^ of hippocampus of the same area was retrieved, and the cortex was first fixed in 2.5% glutaraldehyde followed by 1% osmium tetroxide. The sample was dehydrated through grade concentration of ethanol and soaked in epoxy resin and embedded and, then, sliced using the LKB ultrathin slicer followed by lead staining. The H-600-4 transmission electron microscope was used to observe the ultrastructural changes in the hippocampal cells.

### 2.8. The Brain Histopathological Damage (HD) Assessment

Optical microscopy and electron microscopy were performed to assess the HD scores according to the standards by Laurence Katz. Using the three-grid method, all the neurons (normal and ischemic) of all sides of the respective brain region were counted (in a range of 0.25 × 0.05 mm at a magnification ×200). The proportion of ischemic neurons in each brain region was represented as a percentage of the total neuron count of the region. The average HD score of the ischemic neurons from the five different cerebral regions in each brain tissue section was obtained. The rate of immunohistochemical expression of TNF-*α*, IL-1*β* in the neurons from the hippocampus (CA1-3) was statistically analyzed. Under three random view slides using the three-grid counting method, all positive and negative staining neurons (normal and ischemia) from each side of the corresponding brain regions were counted (0.25 × 0.05 mm range at ×200 magnification), hence determining the percentage of positive staining cells. The number of cases in nonanticoagulation and heparin groups still surviving 72 h after successful CPR was recorded.

### 2.9. Western Blotting

An equal amount (60 *μ*g) of the total protein was separated by SDS-polyacrylamide gel electrophoresis (SDS-PAGE) (Beyotime Biotech, China) and then transferred onto a polyvinylidene difluoride (PVDF) (Pell, USA) membrane after electrophoresis. The membranes were immunoblotted with Abs against CD40, p-p65, and GAPDH (human monoclonal antibody, Sigma, USA) followed by a horseradish peroxidase-conjugated secondary. The immunoblots were detected by using a Bio-Rad Calibrated Densitometer.

### 2.10. Real-Time PCR Analysis

Real-time PCR was performed according to the manufacturer's instructions using the ABI 7300 real-time PCR system with the SYBR Green method. Relative abundances of IL-1*β*, IL-6, and VCAM-1 mRNAs from brain tissues were normalized to the expression level of GAPDH. All amplification reactions were performed in triplicate.

### 2.11. Statistical Analysis

SPSS11.5 was used for the statistical analysis. The data were expressed as the mean ± SD. The differences among the groups were compared using a one-way ANOVA. Unpaired Student' *t*-test was used for direct comparisons. The Kruskal–Wallis analysis was used to compare the differences in the ND and HD scores from different groups. Fisher's exact test was used to compare the success rate of CPR and the 72 h survival rate differences in groups of nonanticoagulation and heparin. Spearman's rank correlation coefficient was used to analyze the correlation between the ND and HD scores of groups of nonanticoagulation and heparin. *P* < 0.05 was considered to be statistically significant.

## 3. Results

### 3.1. Heparin Reduced the Recovery Time after Standard CPR

There was no significant difference between the baseline physiological indexes of nonanticoagulation and heparin groups before the start of asphyxiation (Table 2). After asphyxiation, CA (MABP ≤ 10 mmHg) happened after 167 ± 20 s and 196 ± 32 s in the nonanticoagulation group and heparin group, respectively, manifesting as electrical mechanical dissociation or CA. After standard CPR, it was after 81.3 ± 15.8 and 42.7 ± 20.4 s that the asphyxia-induced CA was reversed, and heparin significantly reduced the recovery time, *P* < 0.01 (Table 3). After CPR, out of the 25 subjects in the nonanticoagulation group, only 16 animals recovered spontaneous respiration successfully, and 9 subjects failed to recover. After CPR, out of the 25 subjects of the heparin group, 23 subjects recovered successfully, while 2 subjects failed. 72 h after ROSC, only 10 out of the 16 subjects from the nonanticoagulation group survived while 6 died out cardiac failure or arrhythmia, whereby in the heparin group, 21 out of the 23 subjects survived while 2 died of heart failure (heart failure or arrhythmia occurred in between 10 and 50 min after ROSC). Therefore, there were, respectively, 10 and 21 subjects from the nonanticoagulation group and heparin group that completed the ND and HD score assessment.

### 3.2. Heparin Alleviated the Brain Damage Induced by Asphyxial CA

There were no anesthesia and vascular catheter used in the control group. Hence, except from weight, ND, and HD scores, there were no other experimental parameters to be observed. The different parameters in the sham group were all stable within the 60 min on life support, with complete recovery of consciousness without any nervous system dysfunction or damage 0.5–1 h after extubation. At the end of the experiment, the ND score for the control group and sham group were 0, respectively, and the overall HD score from the 5 brain regions was also 0 (Tables 4 and 5).

Immediately after ROSC, there was a significant difference in the MABP from the nonanticoagulation group and heparin group, while there was no difference in the other parameters at other time points (Table 2). The time intervals from asphyxiation to CA, from CPR to ROSC, and from mechanical ventilation to extubation were significant different between the nonanticoagulation group and heparin group (Table 3). The heparin group also showed a better CPR success rate of CPR and 72 h survival rate than those in the nonanticoagulation group (Table 3). There was no difference in the ND score among the 4 groups before the experiment and between the control group and sham group at the end of the experiment (Table 4). There was a significant difference in the ND scores 2 h, 24 h, 48 h, and 72 h after ROSC between the nonanticoagulation group and heparin group (Table 4). At the end of the experiment, the HD score for the hippocampus (CA1-3), cortex, thalamic reticular nucleus, lateral shell of caudate nucleus, and cerebella was 0, showing no significant difference among all groups, while there was significant difference between the nonanticoagulation group and heparin group (Table 5). After CA, the nervous system damages in the nonanticoagulation group and heparin group were identified by unilateral or bilateral foot spastic paralysis, but there was no such manifestations in the subjects from the sham group, suggesting that the damage to the nervous system in the nonanticoagulation group and heparin group was, indeed, caused by CA which led to brain damage and not due to the operation. There was a significant correlation between the ND and HD scores of the 2 groups: nonanticoagulation group *r* = 0.86, *P*=0.0018; heparin group *r* = 0.81, *P*=0.0027.

**Figure 1:**
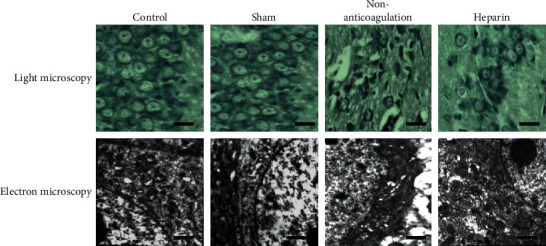
Heparin alleviated the brain damage induced by asphyxial CA. Hippocampus (CA1-3) of SD rats was observed by using the light microscope and electron microscopy from control, sham, noncoagulation, and heparin groups. Scale bar: 50 *μ*m.

After the experiment, the structure of the nerve cells from the hippocampus (CA1-3) collected from the control and sham groups was normal under light and electron microscopy (Figure 1). In the nonanticoagulation group and heparin group, there were different degrees of damage seen in the hippocampus neurons (CA1-3) under a light microscope and electron microscope, the extent of damage being more serious in the nonanticoagulation group (Figure 1).

**Figure 2:**
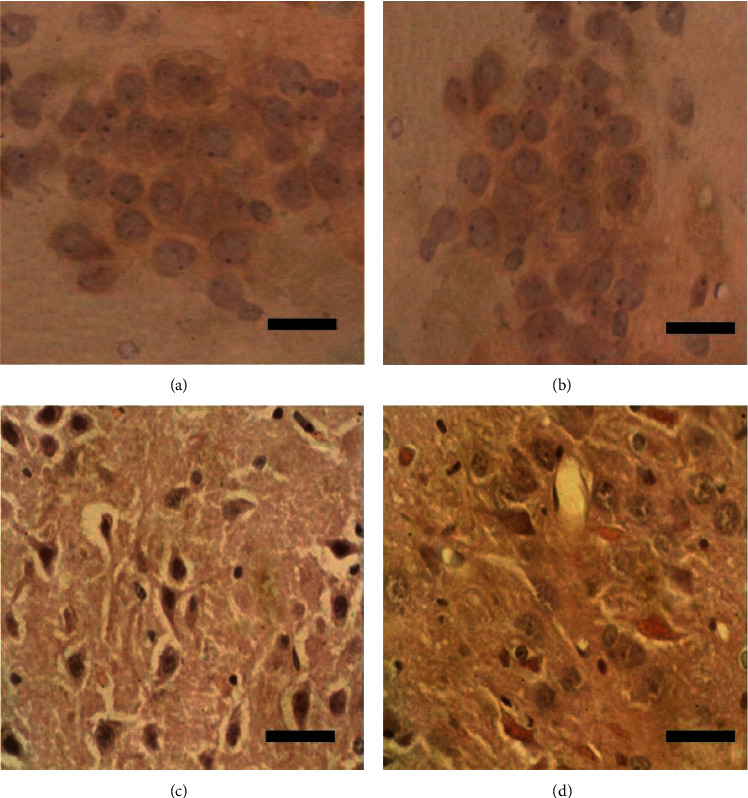
Heparin treatment reduced the expression of TNF-*α* in the hippocampus. Immunohistochemical staining of hippocampus (CA1-3) from control, sham, noncoagulation, and heparin groups is shown. Scale bar: 50 *μ*m. (a) Control, (b) sham, (c) noncoagulation, and (d) heparin.

**Figure 3:**
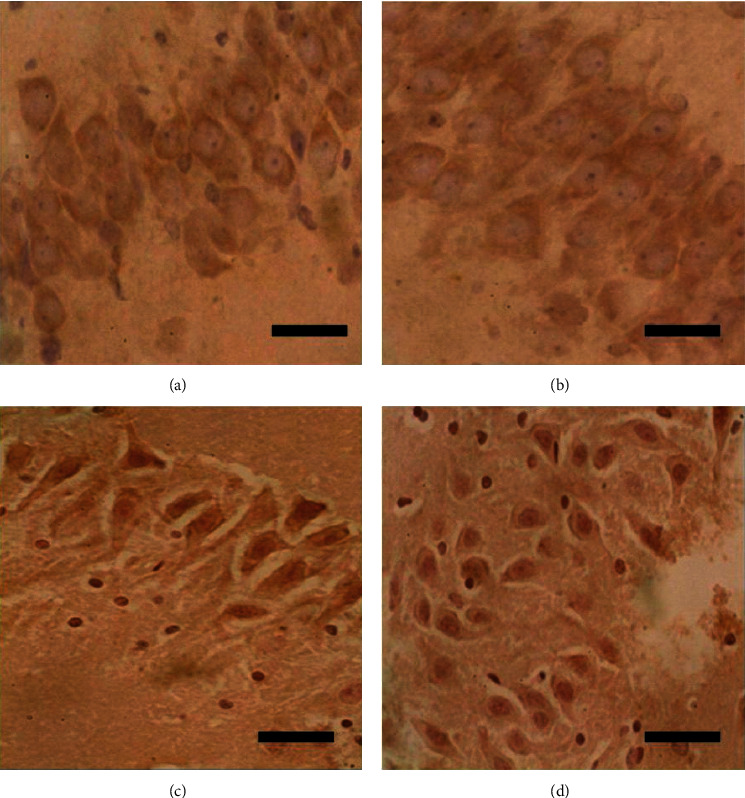
Heparin treatment reduced the expression of IL-1*β* in the hippocampus. Immunohistochemical staining of hippocampus (CA1-3) from control, sham, noncoagulation, and heparin groups is shown. Scale bar: 50 *μ*m. (a) Control, (b) sham, (c) noncoagulation, and (d) heparin.

**Figure 4:**
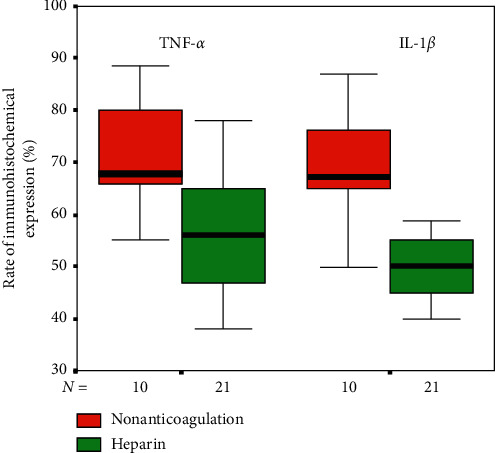
Heparin treatment decreased the percentage of positively stained TNF-*α* and IL-1*β* particles in the hippocampus. The box-plot shows the positive rates of TNF-*α* and IL-1*β* in noncoagulation and heparin groups.

After immunohistochemical staining, there was no positive expression of TNF-*α* (Figure 2) and IL-1*β* (Figure 3) in the hippocampus (CA1-3) of the control and sham groups. However, there was a different extent of positive expression of TNF-*α* (Figure 2) and IL-1*β* (Figure 3) in the hippocampus (CA1-3) of the nonanticoagulation group and heparin group, whereby the rate of positive expression was significantly higher in the nonanticoagulation group. The percentage of positively stained TNF-*α* and IL-1*β* particles in the nonanticoagulation group and heparin group was also significantly different, and heparin treatment significantly decreased the percentage of positively stained cells (Figure 4).

### 3.3. The Protective Effects of Heparin Are Partially Dependent on the Suppression of CD40 and HIF-1*α*

**Figure 5:**
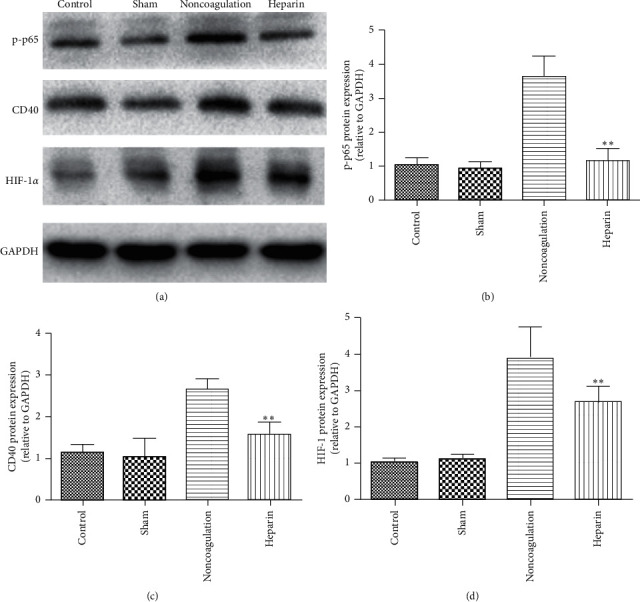
Heparin treatment inhibited the expressions of CD40, NF-*κ*B, and HIF-1*α* induced by asphyxial CA. (a) Representative immunoblots of CD40, p-p65, and HIF-1*α* in the hippocampus. Statistical analysis of CD40 (b), p-p65 (c), and HIF-1*α* (d) expressions. Data are expressed as mean ± SD, *n* = 4. ^*∗∗*^*P* < 0.01 vs. nonanticoagulation.

We, then, tried to find the related signal pathway that was involved in the enhanced inflammatory cytokines. It is well established that the activation of the CD40/CD40L pathway is associated with elevated inflammatory cytokines in a wide range of diseases, such as sepsis, ischemic stroke, and hypertension. Ischemia and hypoxia can also induce the expression of HIF-1*α*, causing major brain damage after CA. So, we collected the brain tissues and detected the expressions of CD40, NF-*κ*B, and HIF-1*α*. As shown in Figures 5(a)–5(d), the expressions of CD40, NF-*κ*B p-p65, and HIF-1*α* were comparable in the brain tissues form control and sham groups. However, asphyxial CA caused a significant increase in CD40, NF-*κ*B p-p65, and HIF-1*α* expressions in brain tissues from the nonanticoagulation group. Treatment of the subject mice with heparin could partially reverse the increased expressions of CD40, NF-*κ*B p-p65, and HIF-1*α*, indicating the inhibitory effects of heparin on CD40/NF-*κ*B and HIF-1*α* pathways. We also used reverse transcriptase PCR to detect some downstream genes of the NF-*κ*B pathway, including IL-1*β*, IL-6, and VCAM-1, in order to show that the NF-*κ*B expression is functionally altered. As expected, their expressions were significantly elevated after asphyxial CA, and heparin significantly inhibited the upregulation of IL-1*β*, IL-6, and VCAM-1.

## 4. Discussion

In this study, we mainly focused on the effects of anticoagulant therapy on the brain function recovery after CPCR and its association with CD40. Based on previous studies, 50–60% of in-hospital deaths after CPR are related to nerve injury, while some are due to irreversible brain damage and residual serious sequelae [15]. Therefore, in a quest to attain maximum possible recovery for the patients, including functional and social recovery has been the focus of attention in the field of medicine and research [16–21]. Even though they can alter cerebral resuscitation prognosis up to some extent, they still cannot manage the microcirculation dysfunction after CA and CPCR. Due to the activation of the coagulation system and inhibition of endogenous fibrinolysis, 5–10 min after CA, there is cerebral microcirculation disturbance and microthrombosis. Therefore, thrombolytic therapy may be a significant improvement in the outcome of cerebral resuscitation, but there is a lack of randomized controlled experiments to support [22]. In line with this hypothesis, our data shows that anticoagulation significantly promoted the recovery of myocardial contractile function, reduced brain tissue damage after CPR, and may eventually improve the prognosis of CPR.

After determining the therapeutic effect of anticoagulation on asphyxial CA-induced brain damage, we tried to find the underlying molecular mechanism by which heparin exerts its protective effects. CD40 protein is a type I transmembrane receptor, belonging to the TNF receptor superfamily. CD40L is an endogenous ligand of CD40, belonging to type II transmembrane protein, TNF superfamily [23]. Indeed, large amounts of research have revealed the substantial role of CD40 in the process of sepsis, atherosclerosis, ischemic stroke, and Alzheimer's disease [9, 11, 24, 25]. Above all, the CD40/CD40L system serves as a vital link between inflammation and thrombogenesis. So, in this study, we collected the brain tissues from all the 4 groups to detect the CD40 protein levels and its downstream NF-*κ*B signaling. As expected, asphyxial CA caused a significant increase in the CD40 expression accompanied by a significant activation of the NF-*κ*B pathway and anticoagulation with heparin before CA could partially reverse the increased CD40 expression and inhibited the activation of NF-*κ*B pathway, suggesting that the effect of heparin to reverse asphyxial CA-induced brain damage is partly dependent on CD40 inhibition.

IL-1*β* and TNF-*α* are the major inflammatory cytokines released by immune cells and local cells in many inflammatory diseases following the activation of CD40 signaling. Our observation indicated that IL-1*β* and TNF-*α* positive staining rates were decreased in brain tissues from the anticoagulation group than that from the nonanticoagulation group, further supporting the involvement of CD40 in heparin-mediated protective effects. The anticoagulation group also showed less damage of neuron compared with the nonanticoagulation group, suggesting that anticoagulation may reduce the expressions of IL-1*β* and TNF-*α*, thus reducing brain damage caused by CA. In our study, we also observed that heparin caused significant changes in HIF-1*α* protein, suggesting that anticoagulant therapy may affect the prognosis of CPR through more complex molecular mechanisms. However, the underlying mechanism is still unclear and needs further research.

Although some meaningful data have been obtained, there are still some limitations in our study. For example, different types of cells in the hippocampus have different tolerance to CA-induced ischemia and hypoxia. Future research will, therefore, focus on understanding the exact molecular mechanisms by which anticoagulants such as heparin combat brain damage caused by CA.

Moreover, complementary and alternative medicine (CAM) is more and more widely used in the treatment of chronic and disabling diseases and is also used for acute diseases during convalescence [14, 26, 27]. CAM tends to be less toxic and less invasive, and CAM is conducive to solving the side effects, environmental pollution, and economic problems of drugs. It has been confirmed by some studies that the combination of Western medicine and CAM therapy is beneficial to the recovery of cerebral and neurological functions in stroke patients [28, 29]. Therefore, future studies should investigate whether heparin combined with CAM can further improve ischemic and anoxic injury of brain cells after CPR.

## 5. Conclusions

In summary, anticoagulation can improve the outcome and prognosis of brain ischemia, including the improvement of neurological function recovery and prevention of morphological and immunohistochemical injury on the brain, while significantly increasing the success rate of CPR. Heparin anticoagulant therapy may improve the prognosis of asphyxial CA by inhibiting IL-1*β*, CD40, NF- B, HIF-1, and other signaling pathways. Our results suggest heparin anticoagulation might be an important supplement to the treatment strategy for the cerebral recovery after CPR.

## Figures and Tables

**Figure 6:**
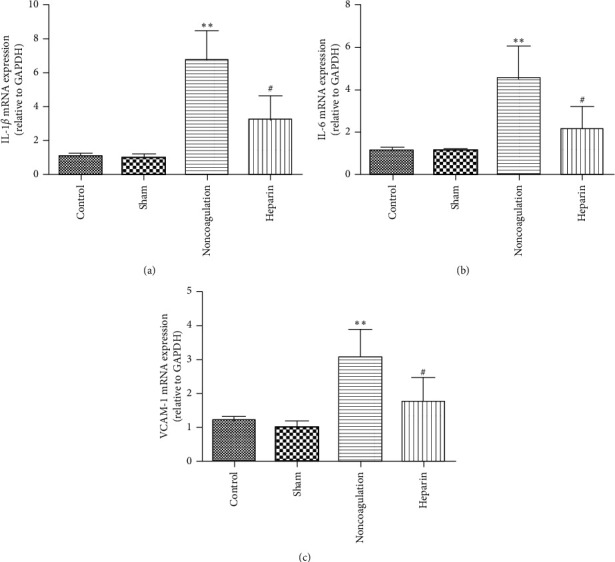
Heparin treatment inhibited the mRNA expressions of IL-1*β*, IL-6, and VCAM-1induced by asphyxial CA. RNA from brain tissues of different groups was analyzed by real-time PCR for IL-1*β* (a), IL-6 (b), and VCAM-1 (c). Data are expressed as mean ± SD, *n* = 4. ^*∗∗*^*P* < 0.01 vs. nonanticoagulation.

**Table 1:** Comparison of the baseline parameters and weights among different groups (mean ± SD).

	Sham (*n* = 10)	Nonanticoagulation (*n* = 25)	Heparin (*n* = 25)
Mean arterial blood pressure (MABP, mmHg)	90 ± 6	89 ± 7	88 ± 8
Heart rate (HR, beats/min)	321 ± 21	319 ± 26	322 ± 24
End-tidal carbon dioxide (P_E_tCO_2_, mmHg)	36 ± 4	36 ± 5	35 ± 5
Fraction of inspired oxygen (FiO, %)	79 ± 3	80 ± 3	80 ± 3
Rectal temperature (RT)	37.9 ± 0.4	38.1 ± 0.6	37.9 ± 0.5
Baseline weight (g)	390 ± 28	387 ± 29	394 ± 27
Weight after 72 h	391 ± 26	343 ± 22^*∗*^	344 ± 1 9^*∗*^

Note: after 72 h, there was significant difference in the weight of in the nonanticoagulation and heparin groups compared with the weight before the experiment, *P* < 0.05.

**Table 2:** Changes in the vitals and other parameters during CPR in nonanticoagulation and heparin groups (mean ± SD).

		Before asphyxiation	ROSC	10 min after ROSC	30 min after ROSC	60 min after ROSC	After ventilation
III (*n* = 10)	MABP (mmHg)	89 ± 7	125 ± 13	96 ± 4	87 ± 6	77 ± 7	77 ± 8
	HR (bpm)	325 ± 23	419 ± 45	349 ± 10	322 ± 24	314 ± 27	286 ± 18
	PEtCO_2_ (mmHg)	36 ± 5	44 ± 4	37 ± 4	33 ± 2	36 ± 3	35 ± 3
	RT (°C)	38.1 ± 0.6	37.5 ± 0.4	37.7 ± 0.4	37.9 ± 0.2	38.2 ± 0.4	38.4 ± 0.3

IV (*n* = 21)	MABP (mmHg)	88 ± 8	145 ± 10^*∗∗*^	98 ± 5	99 ± 7	86 ± 6	79 ± 9
	HR (bpm)	322 ± 19	409 ± 39	358 ± 11	318 ± 21	317 ± 25	289 ± 17
	PEtCO_2_ (mmHg)	35 ± 5	41 ± 5	37 ± 4	34 ± 2	35 ± 2	35 ± 3
	RT (°C)	37.9 ± 0.5	37.7 ± 0.6	37.8 ± 02	37.9 ± 0.1	38.4 ± 0.5	38.5 ± 0.4

Note: there was a significant difference in MABP between the two groups immediately after ROSC, ^*∗∗*^*P* < 0.01, no significant difference among the other parameters.

**Table 3:** The comparison of the CPR success and 72 h survival rates of nonanticoagulation and heparin groups.

	Time from asphyxiation to CA (s)	Time from CPR to ROSC (s)	Time of tracheal extubation (h)	CPR success rate (%)	72 h survival rate (%)
Nonanticoagulation	167 ± 20^*∗∗*^	81.3 ± 20.4^*∗∗*^	6.9 ± 1.4^*∗∗*^	64 (16/25)^*∗*^	62.6 (10/16)^*∗*^
Heparin	196 ± 32	42.7 ± 15.8	4.8 ± 1.2	92 (23/25)	91.3 (21/23)

Note: the measurement data are expressed as mean ± SD, while the count data is expressed as percentages. There is significant difference in the time from asphyxiation to CA and from CPR to ROSC between groups of nonanticoagulation and heparin, ^*∗∗*^*P* < 0.01; the CPR success rate and survival rate 72 hours after ROSC between the two groups were also significantly different, *P* < 0.05.

**Table 4:** The ND scores of groups III and IV at different time points after CPR (mean ± SD%).

Groups	Before anesthesia	2 h after ROSC	24 h after ROSC	48 h after ROSC	72 h after ROSC
Control	0 ± 0	—	—	—	0 ± 0
Sham	0 ± 0	—	—	—	0 ± 0
Nonanticoagulation	0 ± 0	62.9 ± 8.2	39.2 ± 8.3	28.2 ± 5.3	24 ± 7.1
Heparin	0 ± 0	49.1 ± 9.7^*∗∗*^	18.1 ± 6.7^*∗∗*^	4.9 ± 3.9^*∗∗*^	4.5 ± 2.1^*∗∗*^

Note: before anesthesia, the ND score of all the groups was 0 + 0%; there was a significant difference in ND scores between groups of nonanticoagulation and heparin at 2 h, 24 h, 48 h, and 72 h after CPR, ^*∗∗*^*P* < 0.0, 1.

**Table 5:** Ischemic neuron cell count from different brain regions at different time points after ROSC in the 4 groups (mean *t* SD%).

Group	Hippocampus	Cortex	Thalamus	Cerebellum	Shell caudate nucleus
Control	0 ± 0	0 ± 0	0 ± 0	0 ± 0	0 ± 0
Sham	0 ± 0	0 ± 0	0 ± 0	0 ± 0	0 ± 0
Nonanticoagulation	65.7 ± 13.46^*∗∗*^	28.3 ± 7.04^*∗∗*^	30 ± 5.19^*∗∗*^	18.7 ± 3.33^*∗∗*^	18.4 ± 5.62^*∗∗*^
Heparin	26.95 ± 8.42	17.53 ± 6.43	23 ± 4.47	12.74 ± 2.84	12.95 ± 3.73

Note: upon comparing the HD count from the different brain regions, there is a significant difference between the groups of nonanticoagulation and heparin, ^*∗∗*^*P* < 0.01.

## Data Availability

Data can be obtained from the corresponding author upon reasonable request.

## Abbreviations

CA: Cardiac arrest
CPR: Cardiopulmonary resuscitation
CPCR: Cardiopulmonary-cerebral resuscitation
PEtCO_2_: End-tidal carbon dioxide partial pressure
TR: Rectal temperature
MABP: Mean arterial blood pressure
HR: Heart rate
RR: Respiration rate
ECG: Electrocardiogram
EOSC: Restoration of spontaneous circulation
HD: Histopathological damage
ND: Neurological deficit.
